# Production and Characterization of Melt-Spun Poly(3-hydroxybutyrate)/Poly(3-hydroxybutyrate-*co*-4-hydroxybutyrate) Blend Monofilaments

**DOI:** 10.1021/acsomega.4c02241

**Published:** 2024-06-13

**Authors:** Sabrina Kopf, Andrew Root, Ivo Heinmaa, Juliana Aristéia de Lima, Dan Åkesson, Mikael Skrifvars

**Affiliations:** † Swedish Centre for Resource Recovery, Faculty of Textiles, Engineering and Business, University of Borås, 501 90 Borås, Sweden; ‡ MagSol, Tuhkanummenkuja 2, 00970 Helsinki, Finland; § National Institute of Chemical Physics and Biophysics, 12618 Tallinn, Estonia; ∥ Department of Polymer, Fibre and Composite, RISE Research Institutes of Sweden, 504 62 Borås, Sweden

## Abstract

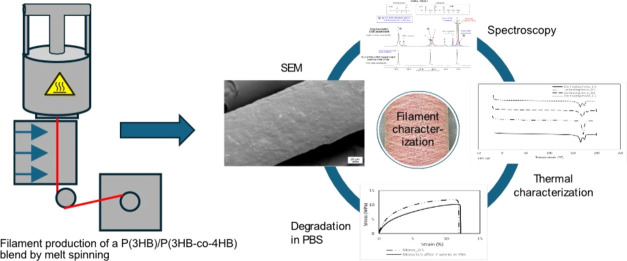

We investigated the melt-spinning potential of a poly(3-hydroxybutyrate)/poly(3-hydroxybutyrate-*co*-4-hydroxybutyrate) blend using a piston spinning machine with two different spinneret diameters (0.2 and 0.5 mm). Results from the differential scanning calorimetry, dynamic mechanical thermal analysis, and tensile testing showed distinct filament properties depending on the monofilaments’ cross-sectional area. Finer filaments possessed different melting behaviors compared to the coarser filaments and the neat polymer, indicating the formation of a different type of polymer crystal. Additionally, the mechanical properties of the finer filament (tensile strength: 21.5 MPa and elongation at break: 341%) differed markedly from the coarser filament (tensile strength: 11.7 MPa, elongation at break: 12.3%). The hydrolytic stability of the filaments was evaluated for 7 weeks in a phosphate-buffered saline solution and showed a considerably reduced elongation at break of the thinner filaments. Overall, the results indicate considerable potential for further filament improvements to facilitate textile processing.

## 1 Introduction

In the multidisciplinary field of tissue engineering, textile technology is one option for producing scaffolds for the replacement of damaged or diseased tissues.^1^ The major benefit of textile technology for scaffold fabrication is its precise placement of yarns, resulting in structural integrity and control of the pore size, both of which are critical characteristics of tissue engineering scaffolds.^2^ The controlled yarn placement enables the creation of scaffolds with controlled anisotropy that mimics the mechanical property of natural tissues.^3^ Besides the scaffold design, the material composition and resulting properties like adsorption in the physiological environment and mechanical performance are important contributing factors to the scaffold’s success.^4^

For biomedical applications, the group of nontoxic and thermoplastic microbial polyesters, polyhydroxyalkanoates (PHAs), have received increased attention.^5^ PHAs are of interest because they show several advantages over the current polymers used, such as poly(lactic acid) (PLA). The main advantages of PHAs are their piezoelectricity, degradation via surface erosion, and degradation products with a higher p*K*_a_ value compared to PLA.^6^ The surface erosion and the higher p*K*_a_ value, which ensure that the pH value of the surrounding tissue remains more stable than in the case of PLA degradation products, indicate the biocompatibility of PHAs,^6^ thus making them more promising as candidates for textile-based tissue engineering scaffolds.

Even though yarns or filaments are the basic components of a textile-based scaffold, to the best of our knowledge, there are yet no commercialized yarns or filaments consisting of PHAs. Melt-spinning of PHA filaments is problematic, especially for poly(3-hydroxybutyrate) (P(3HB)), which is the most common member of the PHA family. Melt-spinning, especially of P(3HB)-based compounds, is problematic for two reasons. First, P(3HB) shows secondary crystallization due to its usually few nucleation points and slow crystallization rates, resulting in large spherulites and material embrittlement.^5^ Second, its melting temperature is close to the thermal degradation temperature.^7^ To overcome these difficulties, the route of microbial production can be changed, and copolymers such as poly(3-hydroxybutyrate-*co*-4-hydroxybutyrate) (P(3HB-*co*-4HB)) can be obtained.^5^

P(3HB-*co*-4HB) is interesting because of its biocompatibility and biosafety.^8^ Depending on the 4-hydroxybutanoic acid (4HB) content, the P(3HB-*co*-4HB)’s mechanical, thermal, and crystalline properties alter.^9^ With increasing 4HB content, the melt and glass-transition temperatures as well as the stress at break decrease, whereas an enhancement of the elongation at break and thermal stability is also observed.^9^ Incorporating an increasing amount of flexible 4HB units in the polymer backbone changes the copolymer from a semicrystalline, brittle material to a ductile, amorphous copolymer at higher 4HB ratios since the 4HB does not cocrystallize in the P(3HB) crystal.^10^

Besides the higher flexibility, increased 4HB content contributes to faster degradation of P(3HB-*co*-4HB) copolymers^11^ because amorphous regions are more prone to hydrolysis compared to crystalline structures.^12^ Poly(4-hydroxybutyrate) homopolymers can have short degradation times of 8 weeks, whereas P(3HB) can take up to 2 years to be degraded.^13^ Therefore, P(3HB-*co*-4HB) copolymers with high 4HB shares are a simple way to reduce the absorption rate and tailor the polymer degradation closer to the healing times of tissues like bone regeneration, which typically takes between 5 and 24 weeks depending on the type of bone.^14^

However, increasing the 4HB comonomer share leads to increased chain flexibility, which results in reduced melt strength and processability of P(3HB-*co*-4HB) with high 4HB shares.^15^ Jo et al. found that blends of amorphous P(3HB-*co*-4HB) (53.7% 4HB) and semicrystalline P(3HB-*co*-4HB) (10% 4HB) show an improved and more constant melt processability compared with a P(3HB-*co*-4HB) copolymer of similar 4HB content.^15^ Hence, polymer blends can be a way to preserve a high 4HB content and biodegradation while improving melt processability.

Several researchers investigated blends of semicrystalline P(3HB-*co*-4HB) and PLA to improve the spinnability and the filaments’ dimensional stability.^16,17^ For biomedical applications, however, PLA might not be the best choice due to its inferior biocompatibility compared with that of P(3HB) or P(3HB-*co*-4HB). Additionally, P(3HB-*co*-4HB)/PLA blends are immiscible in contrast to P(3HB)/P(3HB-*co*-4HB) blends, which only show phase separation at a blend ratio of 50/50 or above.^18^ Therefore, an evenly dispersed blend of semicrystalline P(3HB) and amorphous P(3HB-*co*-4HB) with a high 4HB content could be a promising candidate for melt-spun filaments in biomedical applications. Ideally, the P(3HB-*co*-4HB) would contribute with a reduction of the melting temperature, absorption time, and crystallization, while the P(3HB) enhances the blend tensile strength and melt processability.

The novelty of this research lies in the melt-spinning of filaments from a polymer blend of semicrystalline P(3HB) and amorphous P(3HB-*co*-4HB). The blend consisted of 57 mol % of semicrystalline P(3HB) polymer and 43 mol % of amorphous P(3HB-*co*-4HB) copolymer with 30 mol % of 4HB. Monofilaments were produced using two different types of spinnerets, and their mechanical and thermal properties were characterized. Moreover, the influence of a phosphate-buffered saline (PBS) solution on the filament’s degradation was tested and analyzed. The further aim is to use the made filaments in textile scaffolds for bone cell tissue engineering. Overall, the results indicate great potential for further filament improvements to facilitate textile processing for the intended application.

## 2 Results and Discussion

### 2.1 Polymer Blend Composition

**Figure 1:**
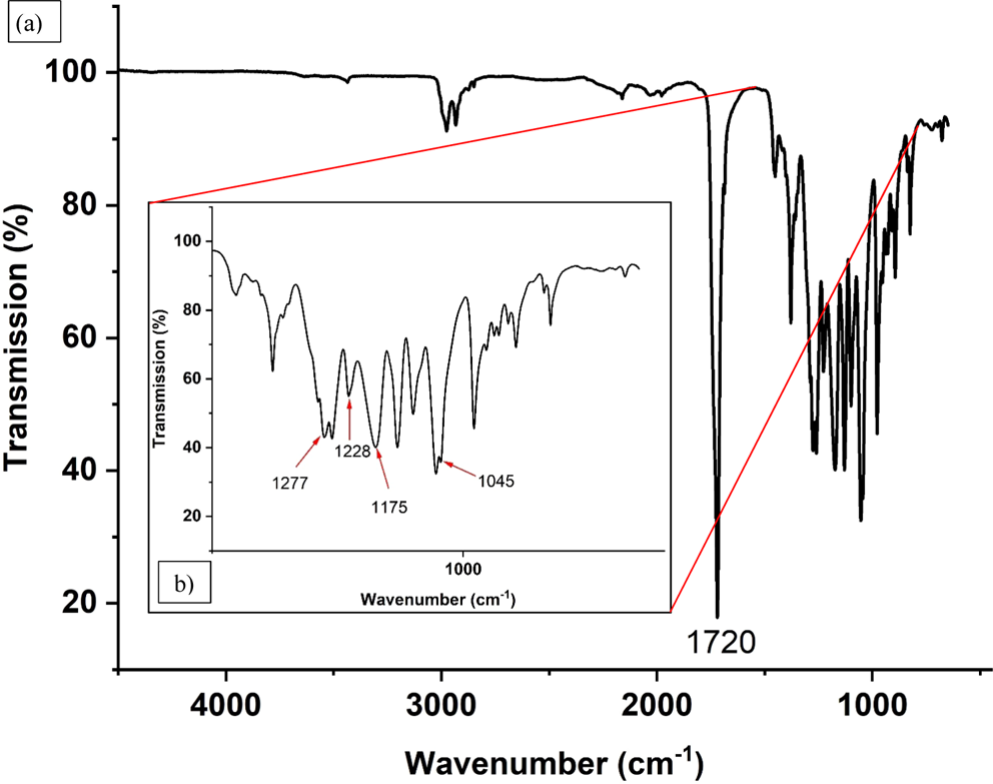
FTIR spectrum of the P(3HB)/P(3HB-*co*-4HB) pellet (a) and a zoom on the fingerprint area (b).

Fourier-transform infrared spectroscopy (FTIR) was used to determine the crystallinity of the used PHA copolymer blend.^19^ Like for most PHAs, the typical stretching vibrations of the carbonyl groups (C=O) are visible at 1720 cm^–1^ in the P(3HB)/P(3HB-*co*-4HB) blend^20^ (Figure 1). The crystalline phase of the polymer blend can be detected at 1277, 1228, and 1045 cm^–1^, whereas the band at around 1175 cm^–1^ is assigned to the C–C stretch in the mobile amorphous phase of the 4HB.^19^ Amorphous phases become more prominent at 4HB contents of 20% and higher.^19^

**Figure 2:**
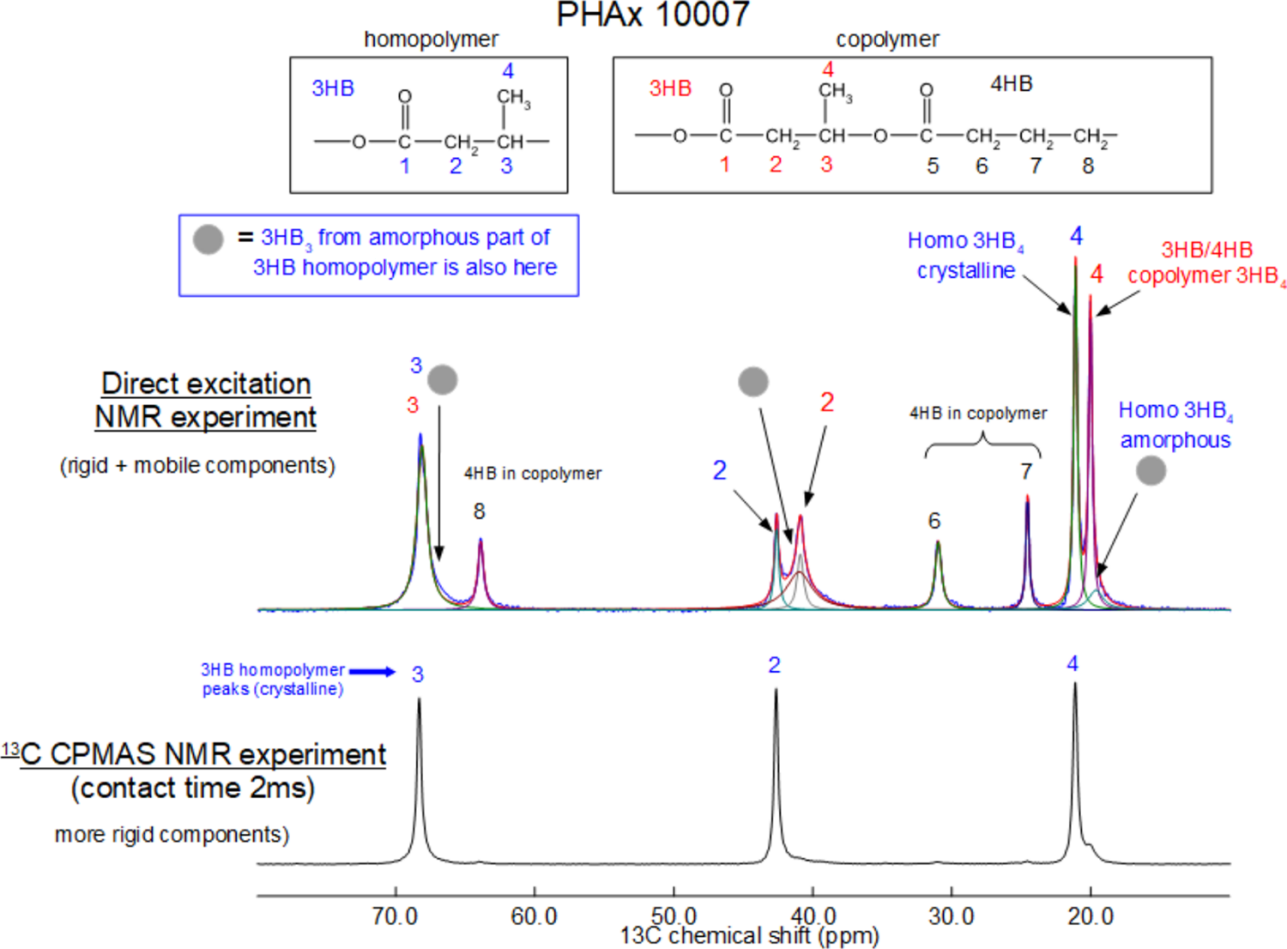
Summary of the PHAx 10007 composition from direct excitation NMR.

^13^C cross-polarization magic angle spinning (CPMAS) and ^1^H MAS nuclear magnetic resonance (NMR) spectroscopy were used to investigate the polymer blend composition in depth. The ^13^C CPMAS NMR spectrum mainly reveals rigid polymer components for which reason mostly strong 3HB peaks were detected, while the 4HB peaks were rather weak, indicating that the 4HB is present in a very mobile phase. During the direct excitation NMR, the 4HB peaks are clearly visible, additionally supporting the high mobility of the 4HB. Further, an expansion of the sample’s carbonyl area was detected, indicating diad structures from a 3HB and 4HB copolymer leading to the conclusion that the polymer blend consists of a P(3HB) homopolymer and a P(3HB-*co*-4HB) copolymer (Figure 2).

The copolymer ratio was calculated based on the direct excitation experiment. In direct excitation experiments, CH_2_ carbons in amorphous 3HB/4HB copolymers have short ^13^C *T*_1_ values and can be used for quantitation.^21^ For the crystalline 3HB homopolymer only, the ^13^C *T*_1_ values for the methyl groups of the 3HB homopolymer are short enough to be used for quantitation of the 3HB homopolymer part of the polymer.^22^ For the estimation of the amorphous 3HB content, the ^13^C CPMAS NMR experiments are used since only the 3HB homopolymer is observed. Finally, the calculations showed a polymer blend with 57 mol % P(3HB) and 43 mol % P(3HB-*co*-4HB) whereupon the P(3HB-*co*-4HB) is made of 30 mol % 4HB.

### 2.2 Filament Extrusion

To the best of our knowledge, it was the first time that a blend of P(3HB) and P(3HB-*co*-4HB) was melt-spun into monofilaments. Two trials were conducted, using two different spinnerets, 0.2 and 0.5 mm, respectively. The spinnability varied greatly depending on the spinneret type. While it was unproblematic to spin and wind the filaments produced with the 0.5 mm spinneret (mono_0.5) at 173 °C (piston drive 1.33 cm^3^/min), it was more challenging for the thinner filaments (mono_0.2). To not exceed the maximum pressure at the mono_0.2 filament production, it was necessary to increase the melt temperature to ca. 178 °C while lowering the piston drive speed (0.14 cm^3^/min), thus increasing the overall residence time. Exposing the polymer for an extended period to temperatures close to its degradation temperature enhances polymer degradation, which was visible after ca. 30 min through a drastic decrease in melt viscosity and drop in the melt pressure, which made it impossible to continue with filament spinning.

### 2.3 Filament Diameter

Choosing a suitable fiber diameter to obtain a desired cell behavior is influenced by several factors such as the type, size, and shape of the cells as well as the scaffold material and fabrication process.^23^ Many studies, especially with electrospun scaffolds, showed that alterations in the fiber diameter change the cell morphology, as well as the cytoskeletal and focal adhesion arrangements.^24^ According to Kun et al., cells can organize themselves around fibers with diameters smaller than the cell.^25^ Considering an intended application in bone tissue engineering, the filament size should be smaller than the size of the main cells involved in bone healing. Bone can heal through primary or secondary healing, which involve different cell types.^26^ However, the majority of clinical relevant fractures heal by secondary healing; thus, the major cell types involved are inflammatory cells, mesenchymal progenitor cells, endothelial cells, chondrocytes, osteoblasts, and osteoclasts.^26^ Cell sizes are around 10–30 μm for most cells like macrophages^27^ or human mesenchymal stromal cells;^28^ however, there are also bigger cells like osteoblasts (20–50 μm)^29^ and osteoclasts (10–300 μm).^30^ When comparing the morphology of cells growing on a nanofibrous and a flat substrate, it is often observed that the cell’s morphology changes based on the substrate’s morphology.^31^ Thus, it seems likely that the cell morphology will also be influenced by filament diameters in the micrometer area. The filaments’ diameter was measured with an optical microscope at a minimum of 80 different locations of the filament. Mono_0.2 showed an average diameter of 88.55 ± 9.24 μm, and mono_0.5 is with 251 ± 25 μm more than 2.5 times bigger than mono_0.2, which is basically the factor in between the two spinneret sizes. Our filaments have a rather coarse diameter (around 86 and 251 μm). Osteoclasts are big cells that could probably grow around the filament. However, the other cells involved in bone healing would probably show a morphology similar to that on a flat substrate because the filament diameter is too large in comparison to the cell size. Therefore, a cell study should be conducted to obtain clarity on how the cells will interact with the filaments.

### 2.4 Thermal Stability of P(3HB)/P(3HB-*co*-4HB) Filaments

Thermal decomposition and stability are important factors affecting the processing of the polymer blend, especially in the piston spinning machine, where the polymer is exposed to elevated temperatures for an extended period. Thus, the polymer pellets were exposed for 2 h to 180 °C where they only showed a mass loss of 0.6%, whereof most of the mass loss was generated within the first 10 min of the isothermal state. This is most likely because of moisture release during polymer drying, as the pellets were not dried prior to the thermal gravimetric analysis (TGA) measurement. Temperatures above 180 °C strongly affect the polymer blend as shown by Luo et al., who observed a weight loss of around 50% after 1 h at 200 °C for a 50/50 polymer blend of P(3HB) and P(3HB-*co*-4HB).^18^ Besides, the researchers showed that a reduction of the P(3HB-*co*-4HB) share further decreased the thermal stability of the blend because pure P(3HB) has a lower thermal stability (5% weight loss at 235 °C) as compared to P(3HB-*co*-4HB) with a 5% weight loss at 254 °C.^18^

Experiments regarding the nonisothermal thermal stability of the filaments at a rate of 10 °C/min were conducted in triplicate, and the average results of the 5 (*T*_5_) and 10 (*T*_10_) percent weight losses and the average peak temperature of the first derivate (*T*_P_), i.e., the maximum decomposition rate, are shown in Table 1.

**Table 1:** Thermal Degradation of P(3HB)/P(3HB-*co*-4HB) Blend Fibers ± Standard Deviation

sample	*T*_5_ (°C)	*T*_10_ (°C)	*T*_P_ (°C)
pellet	265 ± 1.1	271 ± 1.1	288 ± 0.5
mono_0.2	262 ± 4.0	268 ± 3.8	285 ± 2.9
mono_0.5	266 ± 2.8	272 ± 2.6	287 ± 1.7
mono_0.5 rate 0.5 °C/min	191	194	207

**Figure 3:**
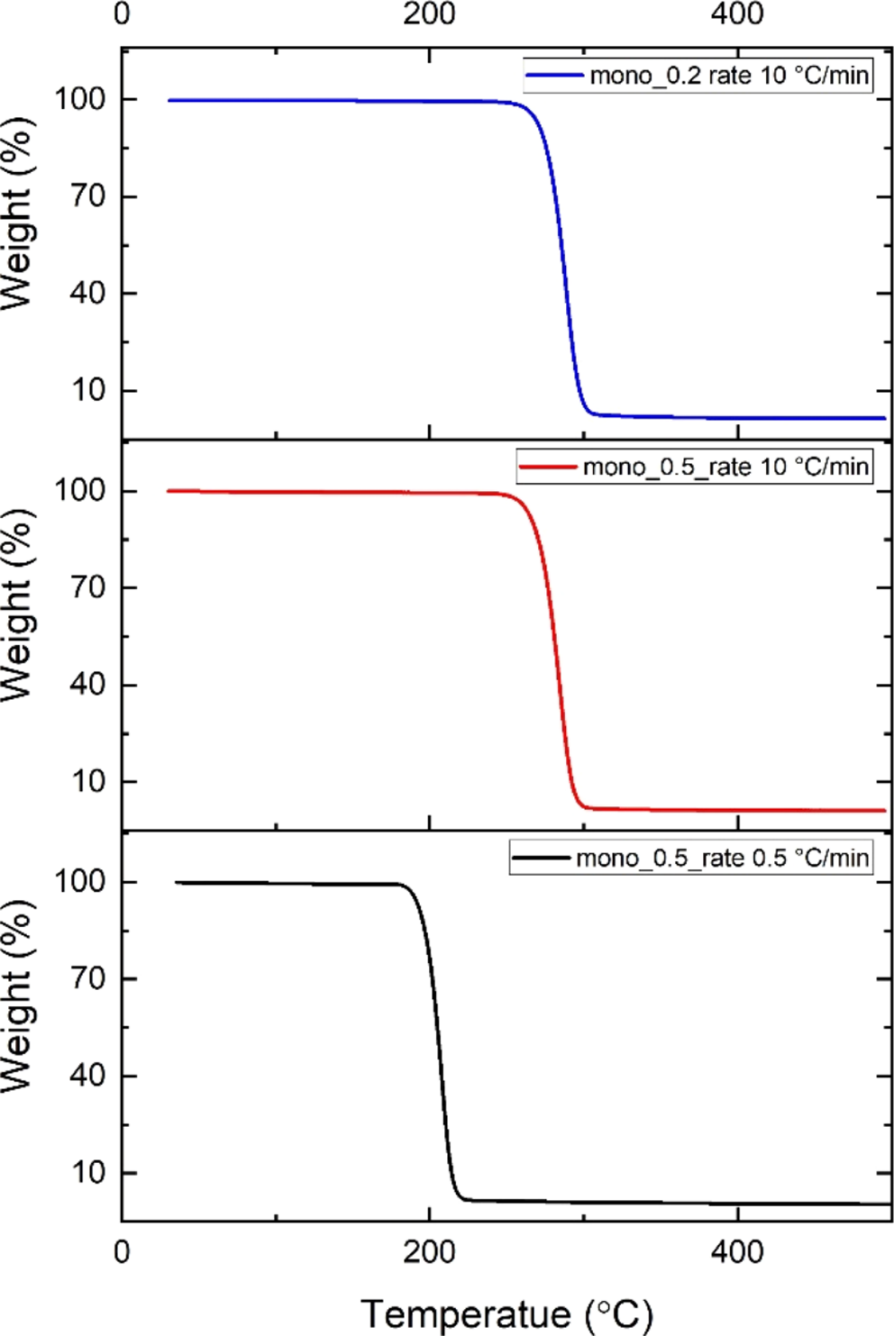
Thermal degradation of a P(3HB)/P(3HB-*co*-4HB) blend at 10 and 0.5 °C/min.

All filaments showed a single degradation step, indicating the miscibility of the polymers. However, the heating rate of 10 °C/min could have been too high to differentiate possible close thermal decomposition temperatures of P(3HB) and P(3HB-*co*-4HB). Thus, an additional test was conducted with a heating rate of 0.5 °C/min, showing again only one single degradation step (Figure 3). This could be an indication for the formation of a polymer inclusion complex even though it seems unlikely.^18^ The slower heating rate adversely affected the thermal stability of the polymer blend, as the comparably long exposure to heat led to a 5% weight loss at 191 °C and a 10% weight loss at 194 °C with a *T*_p_ at 207 °C, which is 80 °C lower than for the filaments heated at a rate of 10 °C/min. A reduction in thermal stability at lower heating rates was also observed by Luo and co-workers and Omura et al., showing that this is a typical behavior for P(3HB) and P(3HB-*co*-4HB) irrespective of their processing history. However, the results at the lower heating rates also indicate that the polymer blend has a limited suitability for slow polymer processing where the polymer is slowly heated to the melting temperature.

The temperature of 5 and 10% weight losses of the filaments is similar to that of the polymer pellets, indicating that the processing did not negatively influence the thermal stability of the fibers. The filaments in this study show not only better thermal stability as compared to blend fibers of PLA and P(3HB-*co*-4HB) (50/50), which show a 5% mass loss at 229 °C^16^ but also as compared to other 50/50 P(3HB)/P(3HB-*co*-4HB) blends that showed a *T*_5_ of 241 °C.^18^ According to Luo et al., pure P(3HB) has a lower thermal stability (5% weight loss at 235 °C) as compared to P(3HB-*co*-4HB) with a 5% weight loss at 254 °C.^18^ The polymer blend tested in this article has a comparably high P(3HB-*co*-4HB) content of approximately 43 mol %, which improved the thermal stability compared to Luo et al.’s work as the 5% weight loss ranges between 262 and 266 °C depending on the polymer’s processing history (Table 1). The polymer blend characterized in this study is probably more thermally stable than the previously mentioned PLA/P(3HB-*co*-4HB) blend because of a higher 4HB share in the copolymer (11 mol % for the PLA blend vs 30 mol % in our blend). The P(3HB-*co*-4HB) is degrading via the β-elimination mechanism that causes random chain scission, which can take place easily in the intramolecular part of the 3HB unit as compared to that of the 4HB unit; thus, a higher 4HB share increases the thermal stability of the copolymer.^9^

The derivative weight loss curve is narrow and showed one maximum at 287 °C for the polymer pellets and the mono_0.5 filaments, while it was similarly shaped but with a marginally lower peak at 285 °C for the mono_0.2 filaments. A single peak for the derivative weight loss indicates miscibility of the polymers, as binary blends usually show one derivative peak for each component. The polymer blend used in this study shows improved thermal properties compared to other P(3HB-*co*-4HB) blend fibers such as a 50/50 blend of PLA and P(3HB-*co*-4HB), which showed two *T*_p_’s at 249 °̊̊C and 313 °C for the P(3HB-*co*-4HB) and PLA share, respectively.^16^

### 2.5 Melting and Crystallization Behavior of the Filaments

Thermal transitions and the polymer’s crystallinity are important processing and end-use properties for the final fibers. To investigate the melting point (*T*_m_), heat of fusion (Δ*H*_m_), temperature of crystallization (*T*_c_), and the degree of crystallinity (χ), differential scanning calorimetry (DSC) was conducted.

The polymer blend pellets show a single melting peak (172 °C) in the first heating cycle and one melting peak with a slight shoulder before the main peak at around 169 °C in the second heating cycle. This is in alignment with the melting temperature of P(3HB), which is usually around 172 °C.^32^ Pure P(3HB-*co*-4HB) copolymer with 4HB contents above 16% is amorphous;^15^ thus, the P(3HB-*co*-4HB) with 30% 4HB is also noncrystalline. However, blends of amorphous and crystalline P(3HB-*co*-4HB) with 4HB contents of 14.4–45% show both cold crystallization and melting peaks, with shifts in the peak areas depending on the blend ratio.^15^ This is because the amorphous P(3HB-*co*-4HB) does not affect the randomness of the crystalline P(3HB-*co*-4HB)’s polymer chain.^15^ Transferring this to the semicrystalline P(3HB) and amorphous P(3HB-*co*-4HB) blend of this study, the amorphous P(3HB-*co*-4HB) probably does not greatly affect the crystallization of the P(3HB) but helps to shift the *T*_m_ to lower temperatures. The crystallization temperature and the degree of crystallinity for the P(3HB) share of the pellets are 113 °C and ca. 50%, respectively. The overall crystallinity of the pellets was around 29% for both the first and second heating cycles. Here, it becomes obvious that the P(3HB-*co*-4HB) attenuates the crystallinity of the blend but does not influence the crystallization of the P(3HB).^18^

**Figure 4:**
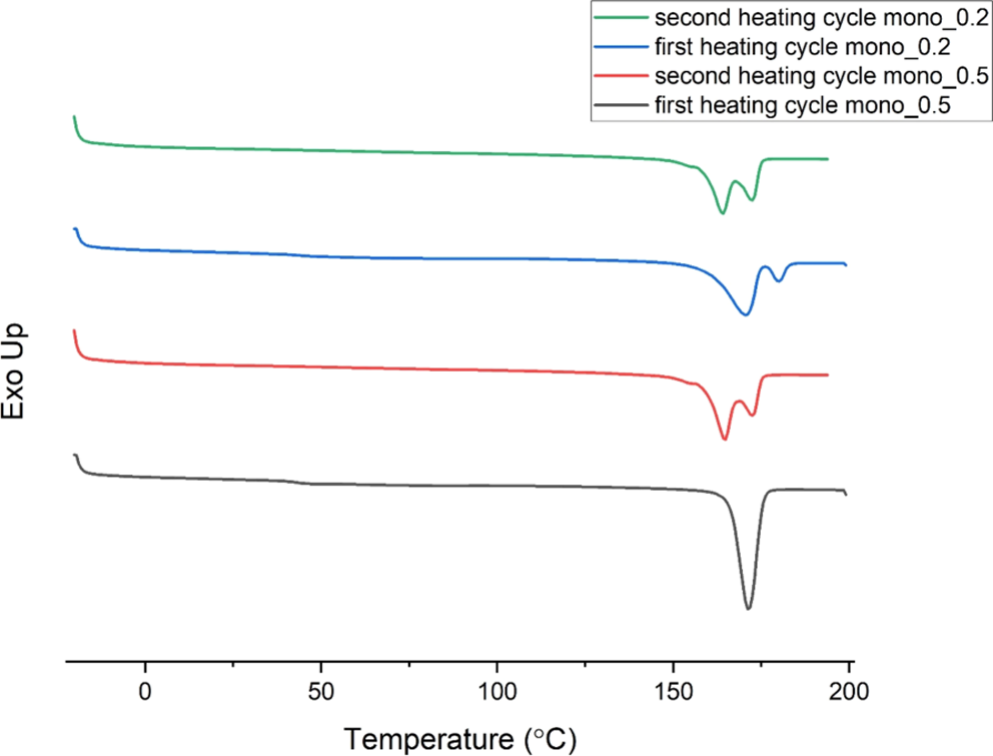
Averaged curves of the first and second melting cycles of mono_0.5 and mono_0.2 filaments.

Monofilaments extruded by the 0.5 mm orifice showed one single melting peak at 171 °C in the first DSC heating cycle and two melting peaks (166 and 174 °C) in the second heating cycle (Figure 4). In contrast, monofilaments produced by the 0.2 mm nozzle showed two melting peaks in their first and second cycles. The difference of the melting behavior probably lies in the slightly different thermal history of the samples.

The melting behavior is strongly dependent on the thermal history of the sample. Melting P(3HB) after isothermal crystallization at *T*_c_ ≥ 120 °C leads to a single melting point, whereas samples solidified at *T*_c_ < 120 °C show two separate melting endotherms.^32^

In melt-spinning, the heat transfer from the filament plays a key role not only for the solidification of the filament but also for the development of the undrawn filament’s internal structure. Even though the processing conditions for the mono_0.2 and mono_0.5 filaments were similar, the die spinneret diameter, which influences the cooling behavior of the filament, was different. Increasing filament diameters lead to an increased relative temperature gradient in the spinning line, which can reach up to 10^3^–10^4^ °C/cm according to calculations by Andrews^33^ and Morrison.^34^ This can result in structural effects in the filament like the formation of crystals along the temperature gradient in, for instance, polyolefin fibers. Furthermore, the crystallinity increases with increasing filament diameter because of the slower cooling resulting in more time for the molecules to form crystalline structures.^35^ A slightly increased crystallinity with increased filament diameter is also noticeable in our samples, especially in the first heating cycle of the DSC measurements (Table 2). Further double melting peaks can originate from a decrease in lamellar thickness or degree of crystallinity.^18^ The additional melting peak of the 0.2 mm filaments in the first heating cycle likely appeared because the polymer chains processed by a smaller orifice experienced a higher reciprocal outflow intensity as compared to the filaments produced by the 0.5 mm spinneret and thus the orientation of these polymers is increased as compared to the thicker filaments.^36^ The oriented polymer chains have different crystallization kinetics and form different crystalline structures probably leading to the additional melting peak.^37^ Alternatively, multiple melting peaks can be formed by melting, recrystallization, and remelting during the material’s heat annealing, where the first peak would represent the crystals generated by cooling.^38^ However, this phenomenon is more often observed for medium-chain length (side-chain length greater than C4) polyhydroxyalkanoates.^39^

**Table 2:** Average Values of the Melting and Crystallization Temperatures of the Neat P(3HB)/P(3HB-*co*-4HB) Blend Pellet (Second Heating Cycle Only) as well as the Monofilaments with 0.2 and 0.5 mm Produced Thereof^a^

sample	*T*_m1_ (°C)	*T*_m2_ (°C)	Δ*H*_m_ (J/g)	*T*_c_ (°C)	χ_blend_ (%)
blend pellet	172 ± 1.5	n/a	41.3 ± 0.2	113 ± 0.2	28.3 ± 0.6
mono_0.2 first heating cycle	171 ± 0.3	180 ± 0.3	43.1 ± 3.2	115 ± 0.06	29.5 ± 2.1
mono_0.5 first heating cycle	171 ± 0.02	n/a	53.7 ± 0.6	115 ± 0.04	36.7 ± 0.4
blend pellet	169 ± 0.3	n/a	43.9 ± 0.5	n/a	29.2 ± 1.0
mono_0.2 second heating cycle	166 ± 0.4	174 ± 0.1	44.0 ± 3.3	n/a	30.0 ± 2.2
mono_0.5 second heating cycle	166 ± 0.01	174 ± 0.03	48.7 ± 0.9	n/a	32.8 ± 1.07

a Additionally, the average degree of crystallinity (χ) for the polymer blend is shown.

For the second heating cycle, both filaments (mono_0.2 and mono_0.5) showed two separated melting peaks at the same temperatures (*T*_m1_ = 166 °C, *T*_m2_ = 174 °C, as shown in Table 2). This is likely because of the comparably fast cooling rate of 10 °C/min, leading to a crystallization below 120 °C. Alternatively, polymer degradation could have happened during the time the pellets spent in the piston spinning machine, leading to phase separation, which is shown as two melting peaks in the DSC. The TGA results indicated that the polymer blend degrades at lower temperatures when exposed to slow heating rates. To clarify the origin of the two melting peaks, size-exclusion chromatography and X-ray diffraction could be done to identify whether a reduction in molecular weight and/or different crystalline structures occurred. This will be studied in our further research.

The degree of crystallinity for the P(3HB) share of the pellets and mono_0.2 filaments was the same in both the first and second heating runs, showing that the filament was not drawn during processing. However, the mono_0.5 filaments were slightly drawn while winding, as reflected in the increased degree of crystallinity. In the first cycle, the mono_0.5 filaments had a crystallinity of ca. 37%, whereas it decreased to almost 33% in the second cycle.

### 2.6 Viscoelastic Properties of the Filaments

**Figure 5:**
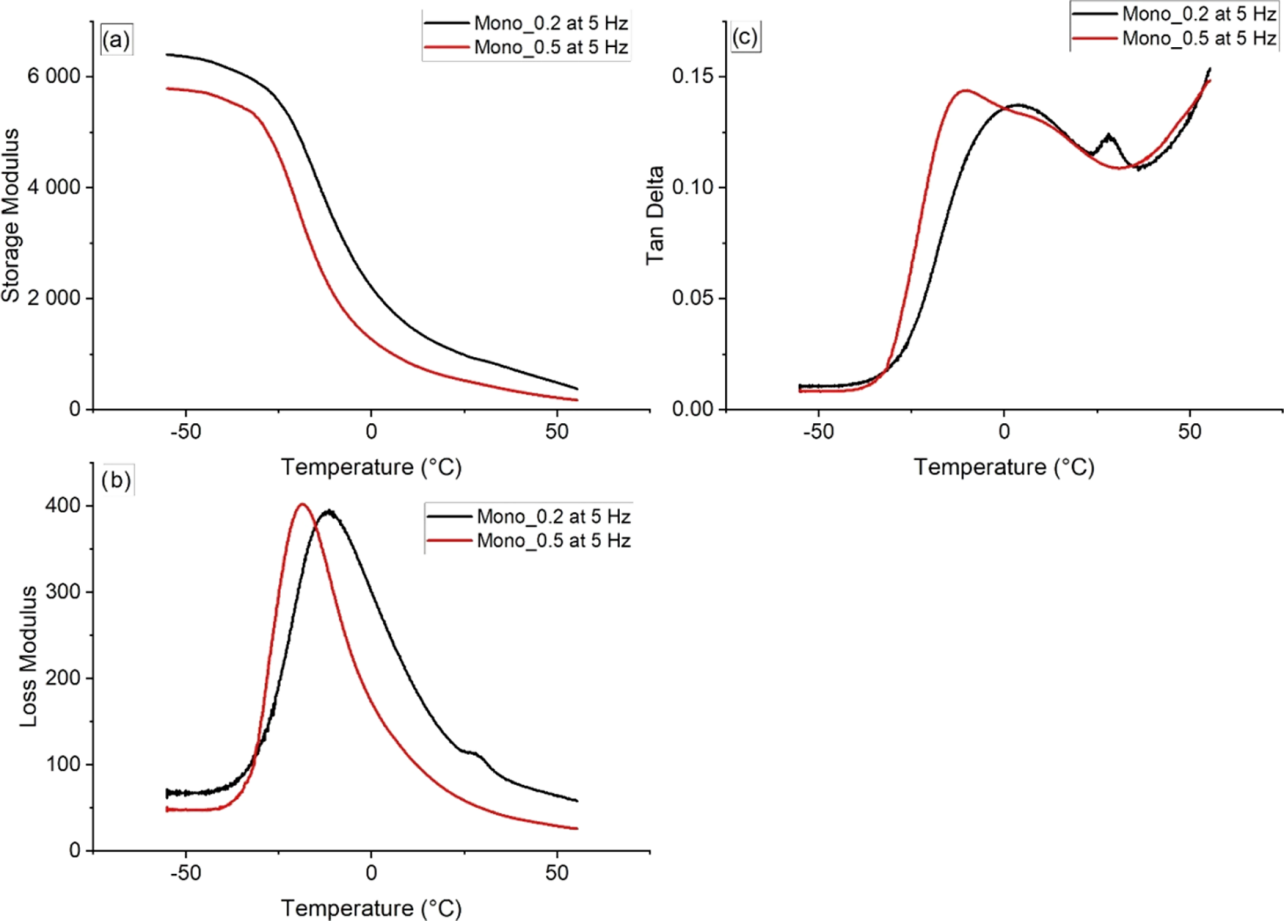
Storage modulus (a), loss modulus (b), and tan δ (c) of the mono_0.5 and mono_0.2 filaments at 5 Hz.

Dynamic mechanical thermal analysis (DMTA) can be used to detect the compatibility of polymer blends, where a binary, incompatible blend shows two platforms in the curve of the storage modulus.^40^ At a frequency of 5 Hz, the P(3HB)/P(3HB-*co*-4HB) blend monofilaments showed a smooth curve in the storage modulus (*E*′) (Figure 5a), indicating a miscible blend. Only a very slight elevation is noticeable at around 30 °C in the monofilaments’ storage modulus, indicating a very slight incompatibility, which is, however, neglectable compared to other compatibilized blends such as a poly(lactic acid)/P(3HB-*co*-4HB) blend, which shows a more distinct step in its storage modulus.^40^

The tan δ curve is used for the determination of the fiber’s *T*_g_. The monofilaments produced with the 0.5 mm spinneret show a broad tan δ peak with one peak and a shoulder on the higher-temperature side at a frequency of 5 Hz (Figure 5c). The elevations indicate the *T*_g_s of both polymers, the P(3HB) and P(3HB-*co*-4HB). The first peak at −11.70 °C is assigned to the P(3HB-*co*-4HB) share of the blend, which are in line with the results of Jo et al., who reported a *T*_g_ of around −15 °C for neat P(3HB-*co*-4HB) with a 4HB content of 35.6%.^15^ The shoulder of the tan δ curve is observed at around 11.30 °C, which presumably represents the *T*_g_ of P(3HB) as it can usually be detected between −4 and +17 °C depending on the polymer and processing method.^5^

Further, the storage modulus shows two slight increases at around −40 and +41 °C (Figure 5a). The slope at −40 °C could indicate a secondary relaxation of the polymer blend. Furthermore, the slope at +41 °C represents most likely the melting of the 4HB-rich crystalline fraction of the P(3HB-*co*-4HB) copolymer.^9^ This is in alignment with the DSC results of Cong et al., who report a melting peak at 41.7 °C for P(3HB-*co*-4HB) with 34 mol % of 4HB.^9^

**Figure 6:**
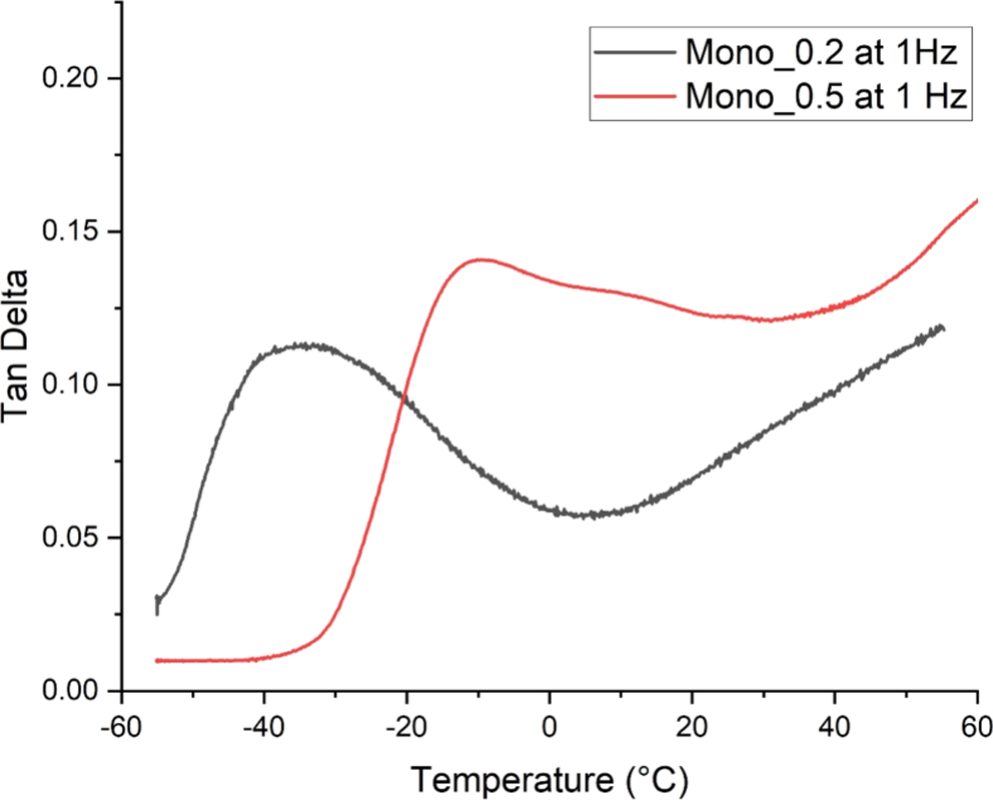
Tan δ of the mono_0.5 and mono_0.2 filaments at 1 Hz.

Fibers produced with the 0.2 mm spinneret showed only one peak at −35.60 °C (Figure 6) in the tan δ curve during the 1 Hz measurement. The single peak for tan δ indicates the miscibility of the polymer blend.

Analyzing the filaments at 5 Hz led to a tan δ with two elevations for the mono_0.5 filament (Figure 5c). Peaks one and two are at −10.95 and 11.45 °C, respectively, comparably close to each other in one common, wider peak area, which ranges from ca. −15 to +30 °C again reflecting the *T*_g_ of P(3HB) and P(3HB-*co*-4HB).

At 5 Hz the mono_0.2 filament showed one smooth curve for the storage modulus, whereas two peaks were visible for both the loss modulus (−11.43 and 28.19 °C) and the tan δ (3.34 and 28.04 °C) (Figure 5). The tan δ of mono_0.2 filament showed one *T*_g_ at 1 Hz, indicating polymer miscibility; thus, it should be possible to apply the Fox equation (eq 1) to predict the new *T*_g_ of the polymer blend.^41^

Fox equation
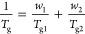
1where *w*_1_ and *w*_2_ represent the weight fractions of components 1 (P(3HB-*co*-4HB)) and 2 (P(3HB)), respectively, and *T*_g1_ and *T*_g2_ are their glass-transition temperatures. Considering a *T*_g_ of −17 °C (determined by DSC) for the P(3HB-*co*-4HB)^20^ at a share of 43% and a *T*_g_ of 2 °C for P(3HB)^42^ at a share of 57%, the predicted *T*_g_ is at 3.85 °C, which is very close to the first peak observed here at 3.34 °C (5 Hz), maybe supporting the assumption of a miscible polymer blend. However, the *T*_g_ is a frequency dependent parameter, and the miscibility assumption was based on the results of the 1 Hz result where the tan δ peaks at ca -35 °C which is not in alignment with the calculations.

### 2.7 Mechanical Behavior of the P(3HB)/P(3HB-*co*-4HB) Filaments

Young’s modulus, which is the ratio of tensile stress to tensile strain, is usually used to compare the mechanical properties of different materials. Hence, the cross-sectional area of the sample is included in the calculation because the tensile stress is the amount of force applied per unit area. Fibers or filaments have very small and irregular (mostly natural fibers) diameters. The specimen’s dimensions such as the cross-sectional area influence the mechanical properties.^43^ For instance, if all other fiber parameters are equal, an increase in the cross-sectional area results in a proportional increase in the fiber’s breaking load.^43^ As compared to the standard tensile test specimen, it can be challenging and time-consuming to accurately calculate the cross-sectional area of each fiber sample. To circumvent this issue, fiber fineness is used in single-fiber tensile testing to be able to compare the fibers’ mechanical properties irrespective of their cross-sectional area.

For more accuracy, the mono_0.5 samples were tested with the single-fiber tensile testing machine. The linear density (fiber fineness) was measured in dtex, which is a unit commonly used in the textile industry and describes the weight of the sample per 10 000 m, i.e., 1 dtex = 1 g/10 000 m. The average linear density of the fibers was 1240 dtex with a minimum linear density of 1179 dtex and a maximum of 1374 dtex, showing that the produced filaments are basically constant in their diameter. The fiber’s mechanical properties are shown in Table 3.

**Table 3:** Average Mechanical Properties of Filaments Spun with a 0.5 mm Spinneret

property (unit)	average value ± standard deviation	minimum	maximum
elongation at maximum force ε_Fmax_ (%)	4.18 ± 0.54	3.07	5.07
maximum force (cN)	108.9 ± 6.17	95.46	118.79
tenacity (cN/tex)	0.88 ± 0.07	0.74	0.98
initial modulus (cN/tex)	41.42 ± 3.83	31.96	47.96

When comparing the tenacity of mono_0.5 with other common textile fibers, it is in the range of elastomers, which also show a tenacity of 0.0088 N/tex.^43^ However, the other mechanical properties are unlike those of elastomeric fibers, especially the elongation and initial moduli. The elongation of our fibers is drastically lower (ε_Fmax_ = 4.18%) compared to the elongation at break of elastomers (>500%), and the initial modulus is much higher with 41.12 cN/tex as compared to 0.0026–0.0071 N/tex.^43^ The mono_0.5 filaments are rather comparable to polyamide 6.6 staple-fibers that show an initial modulus of 0.6 N/tex.^43^

Mono_0.2 filaments could not be tested with the single-fiber tensile tester because the elongation at break was too high, and the machine reached its maximum before the filament could break. Thus, an ordinary tensile tester was used, and 20 samples of the mono_0.5 filaments were tested again for a better comparability of the results.

Table 4 shows the mechanical properties of the P(3HB)/P(3HB-*co*-4HB) filaments. The mono_0.2 filaments reached higher values in tensile strength, elongation at break, and Young’s modulus as compared to the mono_0.5 filaments. This seems surprising as the mono_0.2 filaments were not drawn as compared to the mono_0.5 filaments and had a lower degree of crystallinity, which usually has a strong influence on the tensile properties of a filament.

**Table 4:** Average Mechanical Properties of P(3HB)/P(3HB-*co*-4HB) Blend Fibers ± Standard Deviation

property (unit)	mono_0.2	mono_0.5
tensile strength (MPa)	21.5 ± 2.3	11.7 ± 0.5
elongation at break (%)	341 ± 168	12.3 ± 1.8
Young’s modulus (MPa)	862 ± 160	445 ± 62

Compared to five times hand-drawn monofilaments of P(3HB-*co*-4HB) with 4 mol % of 4HB (tensile strength: 41.9 ± 6.7 MPa, Young’s modulus 150 ± 30 MPa, elongation at break 226 ± 65%), the monofilaments produced in this article have a higher Young’s modulus and the mono_0.2 filaments have a higher elongation at break.^44^ In return, this higher elongation comes with an inferior tensile strength of the mono_0.2 and mono_0.5 filaments as compared to the P(3HB-*co*-4HB) filaments. Almost the same applies when comparing the monofilaments with 2.5 times drawn PLA/P(3HB-*co*-4HB) filaments with a P(3HB-*co*-4HB) share of 45% and a 4HB content of 11%. The PLA blend fibers reach a comparable or lower elongation at break (ca. 12%) but a significantly higher modulus of around 7.5 GPa.^16^

For commercial use, for instance, as a suture, the mono_0.2 and mono_0.5 filaments need further improvement, especially regarding the tensile strength. Commercially available P(3HB-*co*-4HB) monofilament sutures with 16% 4HB have a tensile strength of 167 ± 51 MPa (elongation at break: 113 ± 22 and Young’s modulus: 261 ± 24 MPa).^45^ An improvement of the tensile strength can be achieved by appropriate fiber drawing.

The intended application of our filaments is to be a starting point for textile-based bone tissue engineering. In order to produce a three-dimensional textile scaffold, the filaments need to be processed on knitting or weaving machines where they need to withstand a tensile strength of 500 MPa when being handled on industrial machinery.^46^ To fulfill these requirements, the mechanical properties of the filaments need to be improved, for instance, by fiber drawing. However, the filament’s mechanical properties do not enable us to conclude on the final mechanical properties of the scaffold because the scaffold’s mechanical performance is influenced by the chosen geometry of fabric construction.^47^ Furthermore, the mechanical requirements depend on the type of bone that is intended to be replaced. Trabecular bone has a tensile strength of around 2.25 MPa, whereas the cortical bone has a tensile strength of ca. 125 MPa.^48^ Comparing the tensile results of the obtained filaments (11.7–21.5 MPa) with the tensile strength of bones, it becomes evident that the filaments can compete with trabecular bone (tensile strength: ca. 2.25 MPa) but not with cortical bone (tensile strength: ca. 125 MPa).

### 2.8 Filament Degradation in Isotopic Phosphate-Buffered Saline Solution

**Figure 7:**
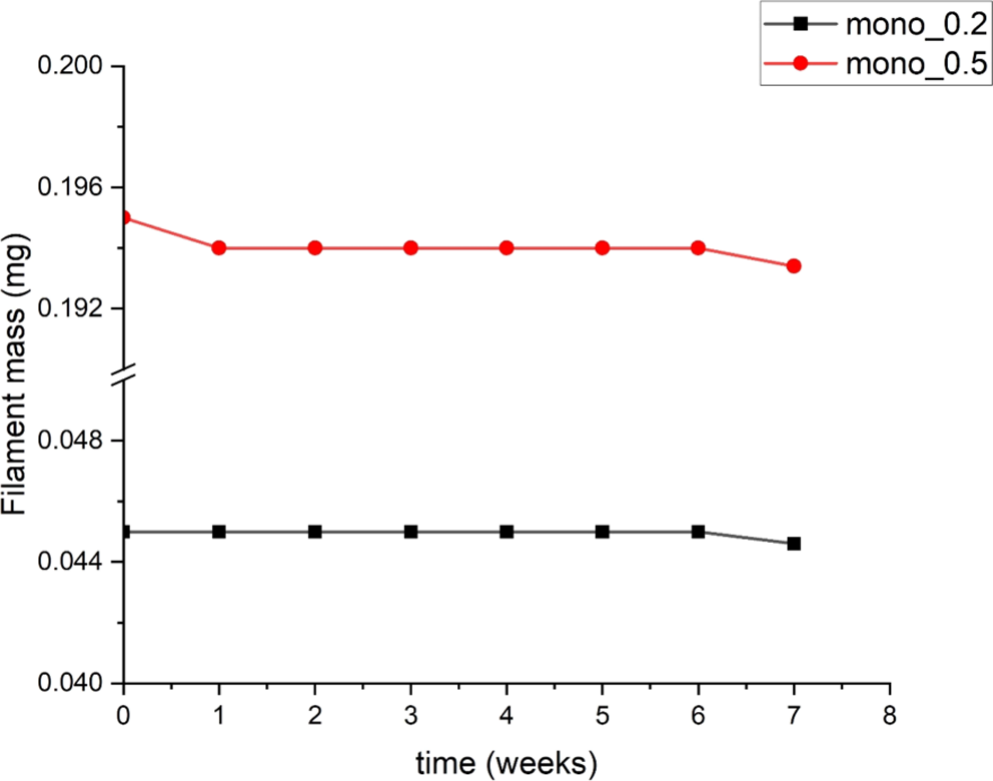
Filament mass over time in phosphate-buffered saline solution for P(3HB)/P(3HB-*co*-4HB) blend filaments.

The filament is intended to be used as a part of a tissue engineering scaffold. Therefore, it is important to get an indication of how the degradation affects the filaments. In general, degradable tissue engineering scaffolds should have the same degradation rate as the regeneration rate of the tissue they replace.^49^ Phosphate-buffered saline (PBS) solution is an isotonic medium often used to simulate the pH of the human body.^50^ Thus, it was used to investigate in vitro whether the filaments would degrade when exposed to physiological media. After being exposed for 7 weeks to PBS, no large weight loss could be detected, neither for the mono_0.2 nor the mono_0.5 filaments, indicating a slow degradation rate (Figure 7). A stable weight in PBS at pH 7.4 is in alignment with the results of Vodicka et al., who did not detect a weight loss for P(3HB) and P(3HB-*co*-4HB) (with 36 mol % 4HB) films in the same time frame.^12^ Protracted degradation could be interesting for bone tissue engineering applications, as ordinary bone fractures usually take 6–8 weeks to heal. Due to the severity of the injuries where bone tissue engineering scaffolds are used, it is supposed that the time for recovery is prolonged as compared to an ordinary bone fracture. Thus, the scaffold should have a higher resistance to degradation compared to an ordinary bone fracture. FTIR scans of the filaments before and after exposure to PBS did not show any chemical modification of the filament’s surface.

**Figure 8:**
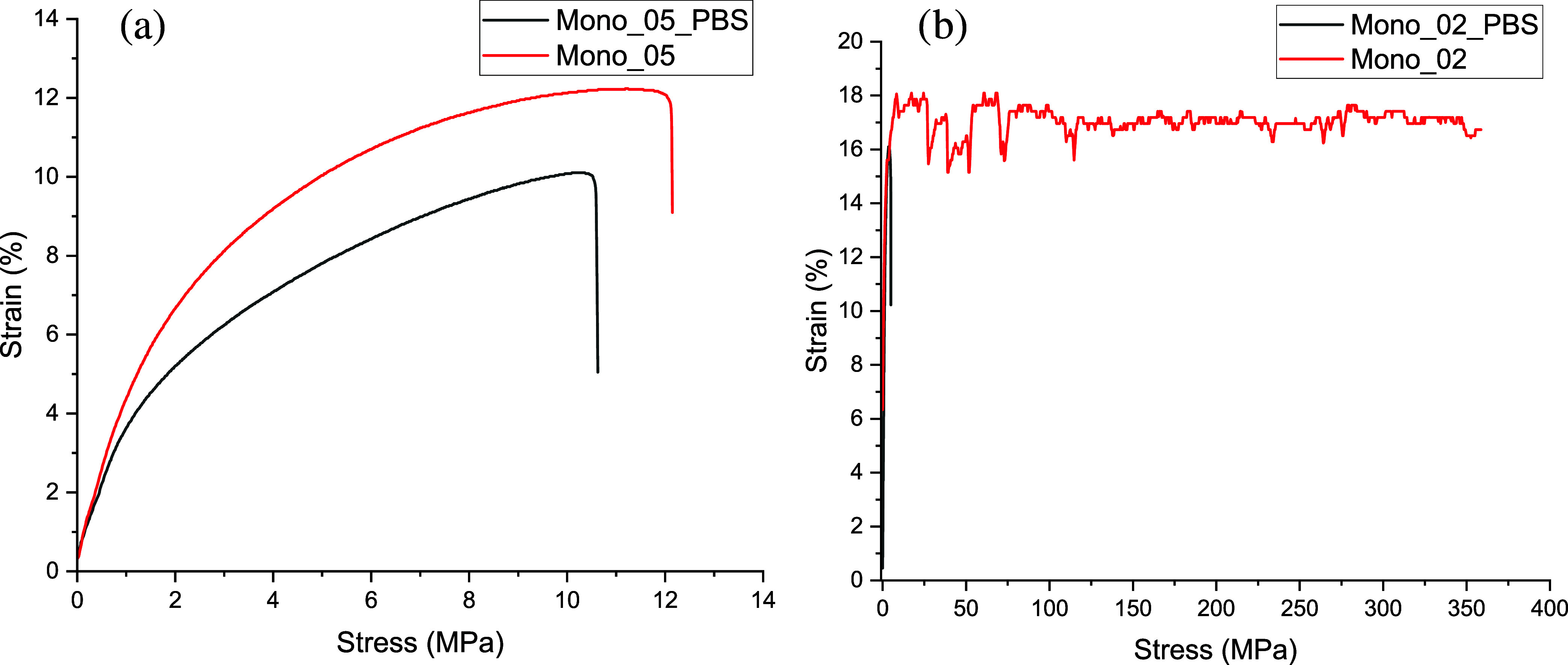
Comparison of the filament’s (a) mono_0.5 and (b) mono_0.2 mechanical properties before and after 7 weeks of immersion in PBS. The graphs shown are selected examples close to the median.

After 7 weeks of immersion in PBS, the mono_0.2 and mono_0.5 filament’s mechanical properties were tested. Prior to tensile testing, the filaments were rinsed with distilled water and dried in a vacuum oven. Both filaments showed a decrease in their elongation at break after exposure to PBS. The mono_0.5 filament’s elasticity decreased slightly from 12.3 to 10.0% after being stored in PBS. On the other hand, the ultimate strength and Young’s modulus increased from 11.71 to 13.63 MPa and from 445 to 563 MPa, respectively (Figure 8).

The thinner filaments showed a pronounced decline in elongation at break from an average of 342% to only 6.7%, which is below the value of the mono_0.5 filaments. On average, the ultimate strength of the mono_0.2 filaments decreased from 21.72 to 16.17 MPa after 7 weeks in PBS, while Young’s modulus increased to some extent (819–997 MPa). The decline in mechanical properties at constant mass is typical for the resorption of biomaterials as it usually starts with water sorption, followed by the reduction of mechanical properties as well as molar mass and ends with weight loss.^6^

**Figure 9:**
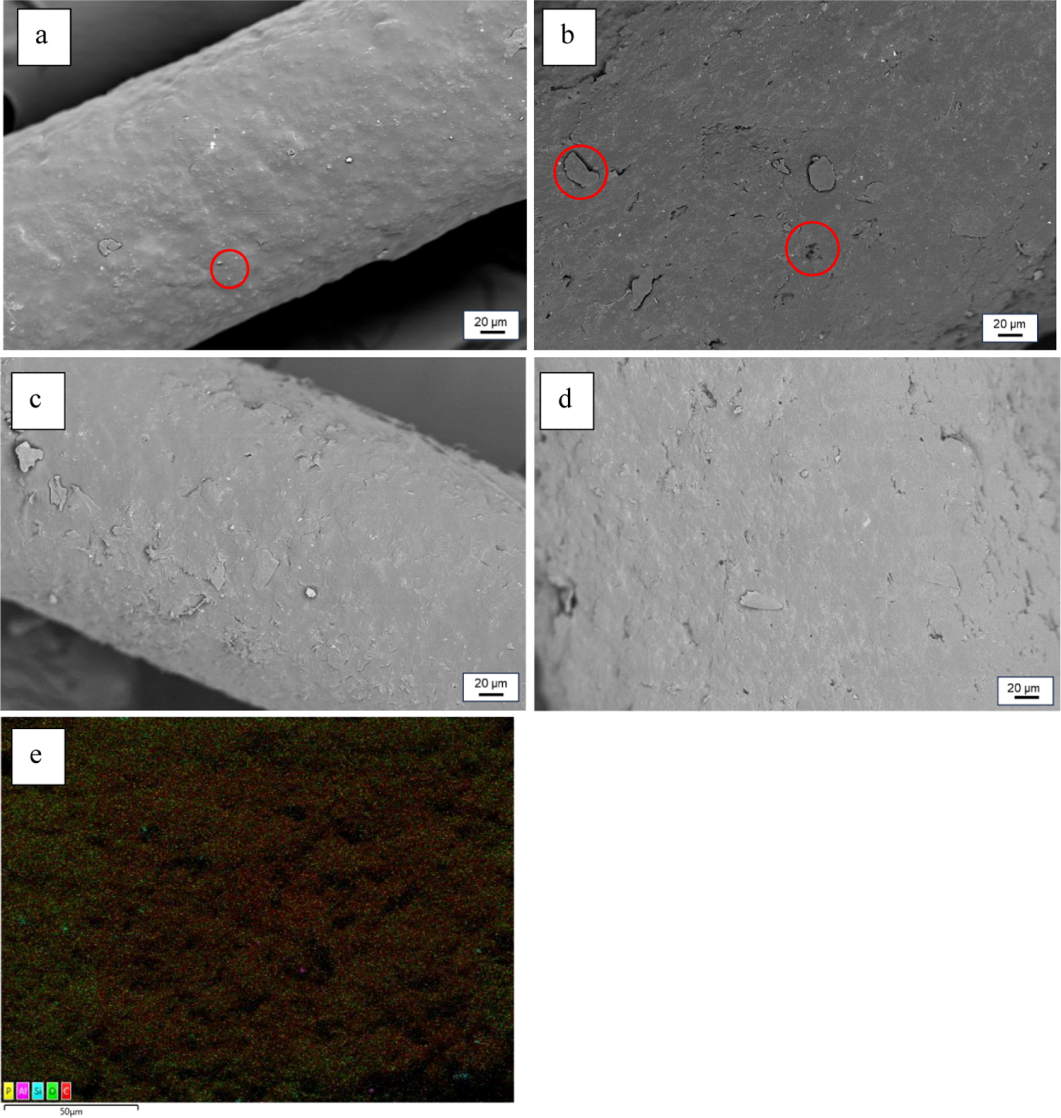
SEM image of mono_0.2 (a) and mono_0.5 filaments (b) before exposure to PBS and after 7 weeks of exposure, (c) mono_0.2 and (d) mono_0.5. Energy-dispersive X-ray spectroscopy (EDS) shows mostly oxygen and carbon of mono_0.5 after 7 weeks in PBS (e).

Both for the mono_0.2 and mono_0.5 filaments, the reduction in elongation is very likely due to the faster degradation of the amorphous areas of the filament’s surface.^51^ The crystalline parts of a polymer provide higher resistance against hydrolysis and are thus degraded at a later state as compared to the amorphous parts in the polymer.^12^ Additionally, the degradation in the amorphous part could reduce the entanglement of the molecular chains and thus help the molecular chains to move more freely and rearrange in a more structured way, increasing the filament’s crystallinity with increasing degradation time.^52^ This assumption is supported by the DSC results, which showed a slight increase in the heat of fusion and thus also in the degree of crystallinity for the mono_0.2 filaments from ca. 30 to 34% in the first heating run. This is in alignment with the results of Vodicka et al., who also found an increase in Δ*H*_m_ for P(3HB-*co*-4HB) samples exposed to artificial body fluids.^12^ The degree of crystallinity of the mono_0.5 filaments basically remained constant. One reason for the decline of tensile properties, indicating degradation but no mass loss, could be that a chain scission of the polymer to shorter chains and oligomers occurs.^12^ These polymer fragments are, like the polymer, rather hydrophobic and thus do not dissolve in the aqueous environment of the PBS but rather adhere to the filament’s surface. Only when the fragments are short enough, they leach into the PBS and are noticeable as a weight loss.^12^ To investigate if the surface structure of the filaments changed, scanning electron microscopy (SEM) images were taken from the samples prior to and after degradation (Figure 9).

Prior to the degradation, the filaments showed a rather smooth surface with scattered droplets/bubbles in the filament (Figure 9a) as well as a few polymer flakes and pits (Figure 9b) on the filament’s surface. To clarify the origin of these irregularities, EDS was conducted, which shows the presence of oxygen and carbon (Figure 9e). This indicates that the irregularities are gas bubbles. It is possible that man-made fibers contain dissolved or dispersed gases, volatile liquids or solids, which are kept in the polymer melt by hydrostatic pressure during extrusion of the polymer.^53^ At the die exit, the pressure drop results in a decreased solubility and trapped gas forms bubbles and/or a pitted surface on the melt-spun filament.^53^ These imperfections reduce the fiber quality and spinnability of the filaments, being one reason for the comparably low mechanical properties of the filaments even before exposure to PBS.

After 7 weeks of exposure to PBS, the surface of the mono_0.5 filament did not change noticeably (Figure 9d), whereas it seems like the surface of the mono_0.2 became rougher and more irregular after being submerged in PBS (Figure 9c). An increase in irregularity with advancing degradation is common for PHAs and is also observed by other researchers.^12,44^

## 3 Conclusions

There is a need to find new material solutions for use in biomedical applications, and PHA polymers are good candidates. PHAs are a versatile group of biopolymers, with chemical composition and properties that can be tailored. Textile scaffolds made from PHA filaments could be used in many biomedical applications if their melt-spinning and further textile processing can be mastered. However, melt-spinning of PHAs, especially P(3HB), is quite challenging due to the narrow processing window, with the melt temperature close to the thermal degradation temperature and the easily occurring secondary crystallization, which leads to material embrittlement. To overcome these challenges, we used a polymer blend of semicrystalline P(3HB) and amorphous P(3HB-*co*-4HB) for melt-spinning of monofilaments. The experimental results showed clearly that monofilaments can be produced with good characteristics and reproducible quality. The finer filaments showed very elastic properties (elongation at break around 300%), whereas the coarse filaments were rather stiff (elongation at break around 12%), which was probably due to a different crystalline structure. The differences in the crystalline structure were verified in the DSC measurements, where the finer filaments showed two melting points, in contrast to the coarser filaments and the unprocessed polymer. A 7 week immersion in a phosphate-buffered saline solution showed a slight decline in the coarser filament’s mechanical properties. In contrast, no weight loss but a drastic reduction in elongation to break and an increased degree of crystallinity was observed for the finer filament. The change in mechanical behavior suggests that some degradation had already occurred. The degradation behavior seems promising for bone tissue engineering applications as the filaments start to degrade within the time span of 7 weeks but do not degrade too rapidly and can therefore still support the growing tissue. Overall, these results show that it is possible to obtain promising filaments from a semicrystalline and amorphous PHA polymer blend. In the future, further improvements regarding their processing and drawing procedures can be made to improve the mechanical performance of the filaments. Additionally, surface characteristics that are important for biomaterials like wettability and cell tests, for instance, cytotoxicity or cell viability, should be studied.

## 4 Materials and Experimental Procedures

### 4.1 Materials

For filament melt-spinning, PHAx 10007 pellets, a blend of semicrystalline and amorphous PHAs were purchased from Helian Polymers (Belfeld, The Netherlands). To characterize the blend composition, Fourier-transform infrared spectroscopy was conducted, followed by solid-state ^13^C nuclear magnetic resonance spectroscopy.

For the degradation study in a phosphate-buffered saline solution, phosphate-buffered saline (0.01 M) in powder form with pH 7.4 for preparing 1 L solutions was purchased from Sigma-Aldrich. The solution was prepared by adding distilled water to the contents of one package.

### 4.2 Filament Preparation

**Figure 10:**
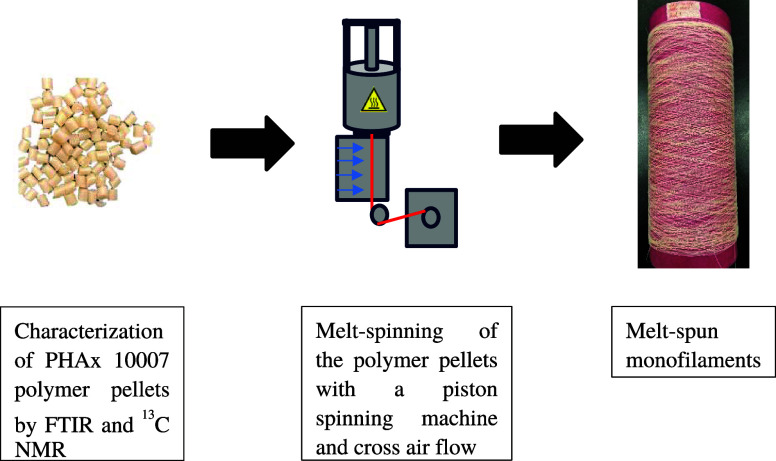
Schematic overview of the material processing steps in this article.

The filament spinnability of the PHA blend was preliminarily tested on a 15 cm^3^ microcompounder (DSM Xplore, Sittard, The Netherlands), where the extruded polymer melt showed a very tacky behavior. Therefore, a 150 cm^3^ piston spinning machine (Fourné Polymertechnik, Alfter, Germany) with an associated controlled cross-flow air quenching system and a bobbin winder was chosen for the filament production. A schematic overview of polymer processing can be found in Figure 10. The cross-flow air quenching system of the piston spinning machine enables a faster and controlled quenching of the filaments. Melt-spinning was carried out using two monofilament spinnerets: 0.2 and 0.5 mm. The monofilaments produced with the 0.2 mm spinneret (mono_0.2) were spun at a melt temperature of 178 °C and a piston drive and melt pressure of 0.14 cm^3^/min and 28 bar. The coarser monofilaments created by the 0.5 mm spinneret (mono_0.5) were produced at a melt temperature of 173 °C at a piston drive of 1.33 cm^3^/min and a melt pressure of 14 bar. Prior to melt-spinning, the heating chamber was filled with nitrogen gas to limit oxidative degradation and the polymer was dried under vacuum for 3 h at 90 °C to remove any moisture. The filaments were air-cooled (25 Pa) and then wound on a bobbin without further drawing on godets. The winding speed for mono_0.5 filaments was 2.3, whereas mono_0.2 filaments were wound at 2.0.

## 5 Methods

### 5.1 Fourier-Transform Infrared (FTIR) Spectroscopy

An FTIR (PerkinElmer Frontier FTIR) was used to analyze the functional groups and crystallinity of the unprocessed polymer pellets by scanning the spectrum 16 times between 450 and 4000 cm^–1^.

### 5.2 Solid-State ^13^C NMR

The blend composition was investigated via nuclear magnetic resonance (NMR) spectroscopy. A solid-state ^13^C NMR was recorded by a Bruker AVANCE-II spectrometer using a set of cross-polarizing magic angle spinning (CPMAS) experiments with varying contact times of 100 μs to 8 ms. A direct excitation magic angle spinning (MAS) NMR experiment was conducted using a relaxation delay of 30 s.

### 5.3 Morphological Characterization

Optical micrographs of the filaments and diameter measurements were acquired by an optical microscope (Nikon Eclipse LV100ND) on 1 m of filament. The filament’s diameter was measured at a minimum of 80 different locations on the coarse and fine filaments. Additionally, the filaments’ surface was characterized by SEM for which reason the filaments were covered in a gold layer and observed by a Zeiss Supra 40 VP SEM using the backscattered electron (BSD) detector at an acceleration rate of 7 kV. Energy-dispersive X-ray spectroscopy (EDS) spectra were acquired by utilizing an EDS detector from Oxford Instruments.

### 5.4 Thermal Characterization of the Filaments

Differential scanning calorimetry (DSC, Q2000, TA Instruments) was used to investigate the degree of crystallinity and melting temperature (*T*_m_) of the produced filaments. The samples were scanned within a range of −20 to +200 °C at heating/cooling rates of 10 °C/min. Before the scan, samples were equilibrated at −20 °C and a nitrogen flow of 50 mL/min was applied during the entire experiment. The melting enthalpy (Δ*H*_m_) of the first heating scan was used to calculate the filament’s degree of crystallinity, whereas the melt enthalpy of the second heating scan was used to calculate the degree of crystallinity for the polymer to investigate if the filament was drawn during production. All calculations were done according to eq 2, where Δ*H*_m_(blend) is the heat of fusion of the blend and Δ*H*_c_(∞) is the 100% crystalline semicrystalline polymer (*P*_sc_ = 146 J/g).

Degree of crystallinity
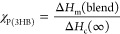
2Thermal gravimetric analysis (TGA) (Q500, TA Instruments, Waters LLC, Wakefield, MA) was conducted to investigate the thermal stability of the polymer pellets and the thermal decomposition of the produced filaments. To investigate whether the polymer blend withstands the prolonged heat exposure of the piston melt-spinning process, the pellets were kept for 2 h isothermal at 180 °C with a nitrogen flow rate of 60 mL/min. For the nonisothermal test, the filaments were heated from 35 to 500 °C at a rate of 10 °C/min under a nitrogen flow rate of 60 mL/min. The temperatures at 5 and 10% mass loss as well as the maximum degradation temperature were determined.

### 5.5 Dynamic Mechanical Thermal Analysis (DMTA)

DMTA is a common method to obtain viscoelastic properties of the monofilament fibers, such as the determination of the glass-transition temperature, the storage (*E*′), and loss modulus (*E*″). In this case, DMTA was used to get an indication of the miscibility of the polymer blend. A temperature sweep was conducted with a DMTA (Q800 TA Instruments) from −20 to 90 °C at a heating rate of 3 °C/min, an amplitude of 15 μm, and a frequency of 1 and 5 Hz using a film and fiber tension clamp.

### 5.6 Mechanical Properties

A single-fiber tensile testing machine was used to test the linear density of the monofilaments prior to tensile testing. The linear density is tested by the vibroscope method, which is common for man-made fibers and based on the vibrating string principle. In a vibroscope, the filament is exposed to a source of sinusoidally alternating energy, which causes the filament to vibrate. The filament’s linear density or mass per unit length can then be calculated from the fundamental resonant frequency of the transverse vibration of the filament under known conditions of length and tension.^54^ Taking the linear density into account gives more accurate results compared to a standard tensile tester because the linear density for each individual fiber is precisely determined and considered in the calculations of the mechanical properties. Thus, 20 samples of the filaments produced with the 0.5 mm spinneret were characterized by a Favimat+ single-fiber testing equipment (Textechno, Mönchengladbach, Germany), which give both the linear density measured by the vibroscope and the tensile strength (tenacity). A gauge length of 20.0 mm was used for both the linear density measurement and the tensile test. For the linear density measurement, a pretension of 0.70 cN/tex and a test speed of 20.0 mm/min were used. The tenacity testing was conducted under a pretension of 0.01 cN/tex and a test speed of 10.0 mm/min. Filaments produced with the 0.2 mm spinneret could not be tested with the Favimat+ because of their extensive elongation, which the Favimat was unable to cover. Thus, the tensile testing for the thinner and coarser filaments was conducted with a Universal H10KT testing machine (Tinus Olsen, Ltd., Horsham, PE) at a crosshead speed of 10.0 mm/min and a gauge length of 20 mm. A caliper was used to measure the diameter of each fiber at three different positions prior to tensile testing. The average fiber diameter was used to calculate the cross-sectional area based on an ideal cylindrical shape, which was then used to calculate Young’s modulus.

### 5.7 Degradation in Phosphate-Buffered Saline Solution

One meter of each filament (mono_0.2 and mono_0.5) was weighed and immersed for 7 weeks at 37 °C in a 0.01 M phosphate-buffered saline (PBS) solution at a pH of 7.4. The solution, prepared by blending a phosphate-buffered saline powder from Sigma-Aldrich (Steinheim, Germany) with distilled water, was changed weekly. Mass changes of the filaments were checked weekly after the samples were rinsed with an excess of distilled water and dried for 12 h at 70 °C in a vacuum oven in order to examine if a mass loss due to degradation occurred.
